# Repeated intranasal esketamine augmentation in treatment-resistant obsessive-compulsive disorder with comorbid major depressive disorder: a prospective case series

**DOI:** 10.1186/s12888-026-08119-5

**Published:** 2026-04-26

**Authors:** Sergi López-Rodríguez, Cinto Segalàs, Eva Real, Mikel Urretavizcaya, Sara Bertolín, José Manuel Menchón, Mª del Pino Alonso

**Affiliations:** 1 Department of Psychiatry, Bellvitge University Hospital, L’Hospitalet de Llobregat, Barcelona, Spain; 2 Neuroscience Program, Bellvitge Biomedical Research Institute-IDIBELL, L’Hospitalet de Llobregat, Barcelona, Spain; 3 Institute of Neurosciences, University of Barcelona, Barcelona, Spain; 4 CIBERSAM, Carlos III Health Institute, Madrid, Spain

**Keywords:** Obsessive–compulsive disorder, Treatment-resistant, Major depressive disorder, Comorbidity, Intranasal esketamine, augmentation

## Abstract

**Background:**

Patients with obsessive–compulsive disorder (OCD) and comorbid major depressive disorder (MDD) represent a severe subgroup with increased treatment resistance, greater functional impairment, and limited therapeutic options. Intranasal esketamine is a rapidly acting treatment for resistant depression, with emerging interest in potential anti-obsessional effects. However, prospective data in this comorbid population remain lacking.

**Methods:**

We present eight adult cases (mean age 47.3 ± 8.8 years) with treatment-resistant OCD (TR-OCD) and comorbid MDD treated at Bellvitge University Hospital. All patients had failed at least two adequate SSRI trials, clomipramine, cognitive-behavioral therapy with exposure and response prevention, and at least one pharmacological augmentation strategy. Intranasal esketamine (56–84 mg/session) was administered according to the standard antidepressant protocol over 12 weeks. Response was defined as ≥ 35% reduction in Y-BOCS and ≥ 50% reduction in MADRS.

**Results:**

Depressive symptoms improved substantially, with MADRS scores decreasing by 48.8% at Week 12 (*p* = 0.0026). Obsessive-compulsive symptoms showed a more modest and heterogeneous reduction, with a 30.3% decrease in Y-BOCS scores (*p* = 0.0037). Four of the eight participants (50.0%) achieved depression response, including two (25.0%) remissions, and four of the eight participants (50.0%) met OCD response criteria. Depressive symptoms improved earlier, whereas OCD symptoms followed a slower and more variable trajectory.

**Conclusions:**

Repeated intranasal esketamine may offer a therapeutic window for patients with severe TR-OCD and comorbid MDD. These preliminary findings support further controlled studies to clarify its role, optimal administration, and integration with psychotherapy.

## Introduction

Obsessive-compulsive disorder (OCD) is a chronic, disabling, and phenotypically heterogeneous condition characterized by intrusive obsessions and repetitive compulsions that markedly impair daily functioning [1, 2]. Despite the availability of established first-line treatments, including high-dose selective serotonin reuptake inhibitors (SSRIs) and cognitive-behavioral therapy (CBT) with exposure and response prevention (ERP), a substantial proportion of patients remain significantly symptomatic after adequate pharmacological and psychotherapeutic interventions, thereby meeting criteria for treatment-resistant OCD (TR-OCD) [3, 4]. Comorbidity is highly prevalent in OCD, and mood disorders are among its most frequent psychiatric associations [5, 6]. In particular, major depressive disorder (MDD) is especially relevant clinically, as depressive comorbidity affects a substantial subgroup of patients with OCD and has been associated with greater obsessive-compulsive severity, broader psychopathology, functional burden, and more complex treatment trajectories [6, 7]. The OCD–MDD intersection is therefore not only common but clinically consequential, especially in patients with severe, refractory illness.

The limited efficacy of purely serotonergic and dopaminergic strategies in a subset of patients with OCD and MDD has increased interest in glutamatergic dysfunction as a shared pathophysiological and therapeutic target [8, 9]. In OCD, converging evidence implicates glutamatergic abnormalities within cortico-striato-thalamo-cortical circuits involved in intrusive thoughts, compulsivity, inhibitory control, and maladaptive habit formation [9]. Consistent with this framework, recent evidence syntheses suggest that glutamatergic agents may have therapeutic potential across obsessive-compulsive and related disorders, particularly OCD, although heterogeneity remains substantial and findings should be interpreted cautiously [10–13]. Glutamate-related mechanisms may also be relevant to treatment processes: neuroimaging data suggest an association between glutamatergic measures, fear extinction, and CBT outcome in OCD [14], pediatric work has linked glutamatergic abnormalities to CBT response [15], and d-cycloserine augmentation of exposure-based CBT has been explored as a strategy to enhance learning-based treatment effects in anxiety, OCD, and related disorders [16].

Against this background, ketamine and esketamine have emerged as particularly relevant glutamate-modulating interventions. Intranasal esketamine has demonstrated efficacy and safety in treatment-resistant depression (TRD) and has become an important addition to the therapeutic armamentarium for refractory depressive disorders [17]. The broader TRD literature further strengthens the rationale for considering esketamine in severe OCD–MDD comorbidity. International expert synthesis has positioned ketamine and esketamine as mechanistically novel options in TRD [18], and more recent review work has summarized both the clinical data and the mechanistic framework supporting their use [19]. Importantly, newer real-world and observational studies provide a richer context for interpreting outcomes. Olivola et al. reported that baseline mentalization and emotional-cognitive rigidity may shape response to esketamine in TRD, a potentially relevant observation in OCD, where rigidity and cognitive inflexibility are clinically prominent [20]. Rosso et al., in data from the REAL-ESK Study Group, showed that clinically meaningful response trajectories may extend into the mid- and long-term course, including later responders, which is especially relevant when different symptom domains may improve at different rates [21]. In addition, Sarasso et al. suggested that esketamine’s therapeutic action may extend beyond conventional symptom counts to experiential and phenomenological dimensions such as disembodiment and affective resonance, broadening the conceptual framework through which its clinical effects may be understood [22].

Within OCD specifically, interest in ketamine- and esketamine-based approaches has grown steadily. Systematic reviews suggest preliminary anti-obsessional effects of ketamine, but also emphasize the small samples, heterogeneous methodologies, and predominance of single-session intravenous studies in the available literature [11–13]. More recently, a retrospective chart review reported preliminary improvement in obsessive-compulsive symptoms with esketamine augmentation in treatment-resistant OCD [23]. However, prospective evidence remains extremely limited, and, to our knowledge, no prior prospective case series has specifically examined repeated intranasal esketamine augmentation in patients with TR-OCD and comorbid MDD. To address this gap, we conducted a prospective case series at Bellvitge University Hospital, a specialized referral center with a dedicated OCD Unit. Eight adult patients with severe TR-OCD and comorbid MDD, all of whom had failed at least two adequate SSRI trials, CBT with ERP, clomipramine, and at least one pharmacological augmentation strategy, and who presented baseline Montgomery–Åsberg Depression Rating Scale (MADRS) scores ≥ 35 and Yale-Brown Obsessive-Compulsive Scale (Y-BOCS) scores ≥ 24 [24–26], received repeated intranasal esketamine (56–84 mg/session) according to the approved antidepressant protocol. We evaluated baseline clinical characteristics, 12-week changes in depressive and obsessive-compulsive symptoms, and overall tolerability, with the aim of generating preliminary data to inform future controlled studies in this highly refractory comorbid population.

## Patients and methods

This study was designed as a prospective observational case series. Owing to its descriptive and hypothesis-generating nature, it should not be interpreted as a controlled efficacy study.

### Study setting and participants

The Psychiatry Department at Bellvitge University Hospital is a recognized referral center in Catalonia for the diagnosis and treatment of adult OCD, evaluating approximately 300 new patients per year from across Catalonia and other regions of Spain. We consecutively recruited eight adult patients between July 2023 and August 2024 who met all the following criteria: (1) a primary DSM-5 diagnosis of OCD with a baseline Yale–Brown Obsessive–Compulsive Scale (Y-BOCS) total score ≥ 24; (2) a co-occurring major depressive episode with a Montgomery–Åsberg Depression Rating Scale (MADRS) score ≥ 35; and (3) inadequate response to at least two adequate trials of selective serotonin reuptake inhibitors (SSRIs), cognitive–behavioral therapy (CBT) including exposure and response prevention (ERP), clomipramine, and at least one pharmacological augmentation strategy.

According to the Pallanti–Quercioli proposal, treatment resistance in OCD can be descriptively staged on the basis of illness severity, prior treatment failures, and degree of refractoriness. In our sample, all patients fulfilled criteria consistent with severe treatment-resistant illness, corresponding descriptively to levels 3–4 of this framework [4].

In this framework, levels 3–4 typically correspond to severe treatment-resistant OCD, characterized by persistent symptoms despite multiple adequate pharmacological trials and structured CBT with exposure and response prevention, generally associated with Y-BOCS scores in the severe range (≥ 24).

### Treatment protocol

Eligible patients initiated intranasal esketamine augmentation to their pre-existing psychopharmacological regimen, with doses ranging from 56 to 84 mg per session according to the approved antidepressant protocol for treatment-resistant depression. The induction phase consisted of two sessions per week for approximately four weeks, followed by once-weekly sessions until Week 12, with minor adjustments based on clinical evolution and clinician judgment. Concomitant medications, including SSRIs and antipsychotics, were maintained throughout the observation period.

All patients had previously undergone CBT including ERP as part of their treatment-resistant status. However, structured psychotherapy was not prospectively standardized, protocolized, or experimentally manipulated during the study period. Therefore, the possible contribution of concomitant or ongoing psychotherapy to clinical outcomes could not be systematically quantified and should be considered an uncontrolled factor.

### Assessments and data collection

**Fig. 1:**
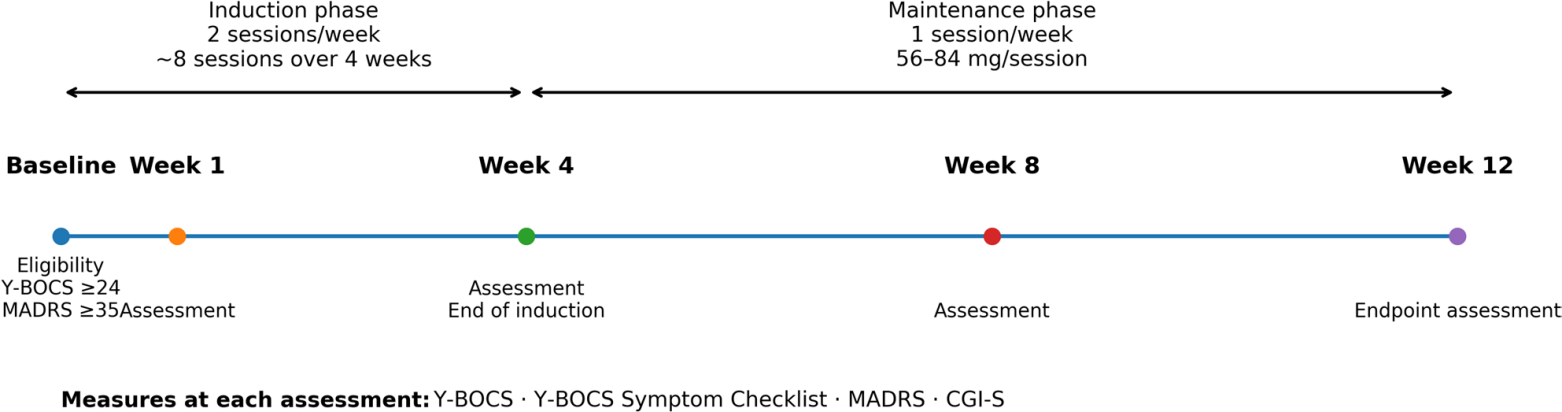
Study timeline, assessment points, and esketamine session frequency. Patients were assessed at baseline and at Weeks 1, 4, 8, and 12 using the Y-BOCS, Y-BOCS Symptom Checklist, MADRS, and CGI-S. Intranasal esketamine was administered according to the standard antidepressant protocol, with an induction phase of approximately 2 sessions/week during the first 4 weeks, followed by approximately 1 session/week until Week 12

Demographic information, OCD resistance level according to the Pallanti–Quercioli proposal, total number of intranasal esketamine sessions, and clinician-recorded adverse events were collected for each patient. Symptom severity and clinical status were evaluated at baseline, Week 1, Week 4, Week 8, and Week 12 (or last observation if dropout occurred) using the Y-BOCS, the Y-BOCS Symptom Checklist, the MADRS, and the Clinical Global Impression–Severity scale (CGI-S). Detailed demographic and baseline clinical characteristics of the sample are presented in Table 1. Figure 1 shows the study timeline, including assessment points and session frequency.

### Response and remission criteria

For OCD, response was defined a priori as a ≥ 35% reduction from baseline Y-BOCS, in line with commonly used criteria for clinically meaningful treatment response in OCD studies [27, 28]. For depression, response was defined as a ≥ 50% reduction from baseline MADRS, and remission as a MADRS total score ≤ 10 at endpoint.

### Statistical analysis

Primary analyses compared baseline versus Week 12 scores for MADRS and Y-BOCS using two-sided paired t-tests. We report mean change (points), 95% confidence intervals, p-values, and within-subject effect sizes (Cohen’s dz). Non-parametric sensitivity analyses were conducted using Wilcoxon signed-rank tests. Statistical significance was set at two-sided *p* < 0.05. No multiplicity correction was applied given the exploratory design and the small sample size (*n* = 8). Analyses were performed using IBM SPSS Statistics for Windows, Version 30.0 (IBM Corp., Armonk, NY, USA).

### Ethics statement

The study was conducted in accordance with the Declaration of Helsinki and was approved by the Bellvitge University Hospital Clinical Research Ethics Committee (Acta 25/24; CEIm entry 2024/2492). All participants provided written informed consent prior to inclusion.

**Table 1:** Baseline demographic, clinical, and treatment characteristics of the study sample (*n* = 8). Values are presented as individual patient data

Id	Sex	Age, years	Main OCD symptoms	Pallanti Severity Index/resistance level	Comorbidities (Medical / Psych)	MADRS wk0	Y-BOCS wk0	CGI-S wk0	Previous treatment strategies	Duration of Illness (years)	Total Sessions	Final Esketamine Dose (mg)	MADRS wk12	Y-BOCS wk12	CGI-S wk12
1	M	49	Contamination	3	Hypertension / Social Anxiety Disorder, MDD	35	24	5	SSRI + Antipsychotic, CBT-ERP	7	12	84	4	16	2
2	F	37	Moral (Scrupulosity)	3	Fibromyalgia / Generalized Anxiety Disorder, MDD	35	28	4	SSRI + TCA, CBT-ERP	10	10	84	5	12	1
3	M	47	Checking	4	MDD	35	32	6	SSRI + BZD, CBT-ERP	4	8	56	20	30	5
4	M	59	Contamination	4	Hypothyroidism / Panic Disorder, MDD	40	36	7	Clomipramine + Antipsychotic, CBT-ERP	8	12	84	22	18	2
5	F	44	Moral (Scrupulosity)	3	IBS / PTSD, MDD	40	28	4	SSRI + Antipsychotic, CBT-ERP	6	6	56	16	16	2
6	F	36	Checking	4	MDD	35	34	6	SSRI + TCA + BZD, CBT-ERP	9	11	84	30	32	6
7	M	60	Checking	4	HT, DLP, MDD	45	35	7	SSRI, CBT-ERP	5	9	56	45	30	7
8	F	46	Contamination	3	Epilepsy, fibromyalgia, MDD	35	24	5	SSRI + Lithium, CBT-ERP	10	5	56	16	14	3

Abbreviations: BZD = benzodiazepine; CBT-ERP = cognitive-behavioral therapy with exposure and response prevention; CGI-S = Clinical Global Impression–Severity; DLP = dyslipidemia; HT = hypertension; IBS = irritable bowel syndrome; MADRS = Montgomery–Åsberg Depression Rating Scale; MDD = major depressive disorder; OCD = obsessive-compulsive disorder; PTSD = post-traumatic stress disorder; SSRI = selective serotonin reuptake inhibitor; TCA = tricyclic antidepressant; Y-BOCS = Yale–Brown Obsessive-Compulsive Scale

## Results

Symptom severity and treatment response were assessed at baseline, Week 1, Week 4, Week 8, and Week 12 (or last observation if dropout occurred) using the Y-BOCS, Y-BOCS Symptom Checklist, MADRS, and CGI-S.

### Temporal pattern of improvement

Clinically, depressive symptoms tended to improve rapidly, with most MADRS reduction observed within the first four weeks and sustained thereafter. In contrast, OCD symptoms generally showed slower and more heterogeneous change: minor reductions were often noted by Week 4, with more consistent improvement emerging between Weeks 8 and 12 in responders.

### Statistical outcomes

From baseline to Week 12, MADRS decreased from 37.5 ± 3.78 to 19.75 ± 13.32 (mean change 17.75, 95% CI 8.53–26.97; t(7) = 4.55, *p* = 0.0026; Cohen’s dz = 1.61). Y-BOCS decreased from 30.13 ± 4.79 to 21.00 ± 8.16 (mean change 9.13, 95% CI 4.22–14.03; t(7) = 4.28, *p* = 0.0037; Cohen’s dz = 1.51). Mean percentage improvements were 48.8% for MADRS and 30.3% for Y-BOCS. Wilcoxon signed-rank tests corroborated these results (MADRS *p* = 0.018; Y-BOCS *p* = 0.0078).

### Response rates

**Fig. 2:**
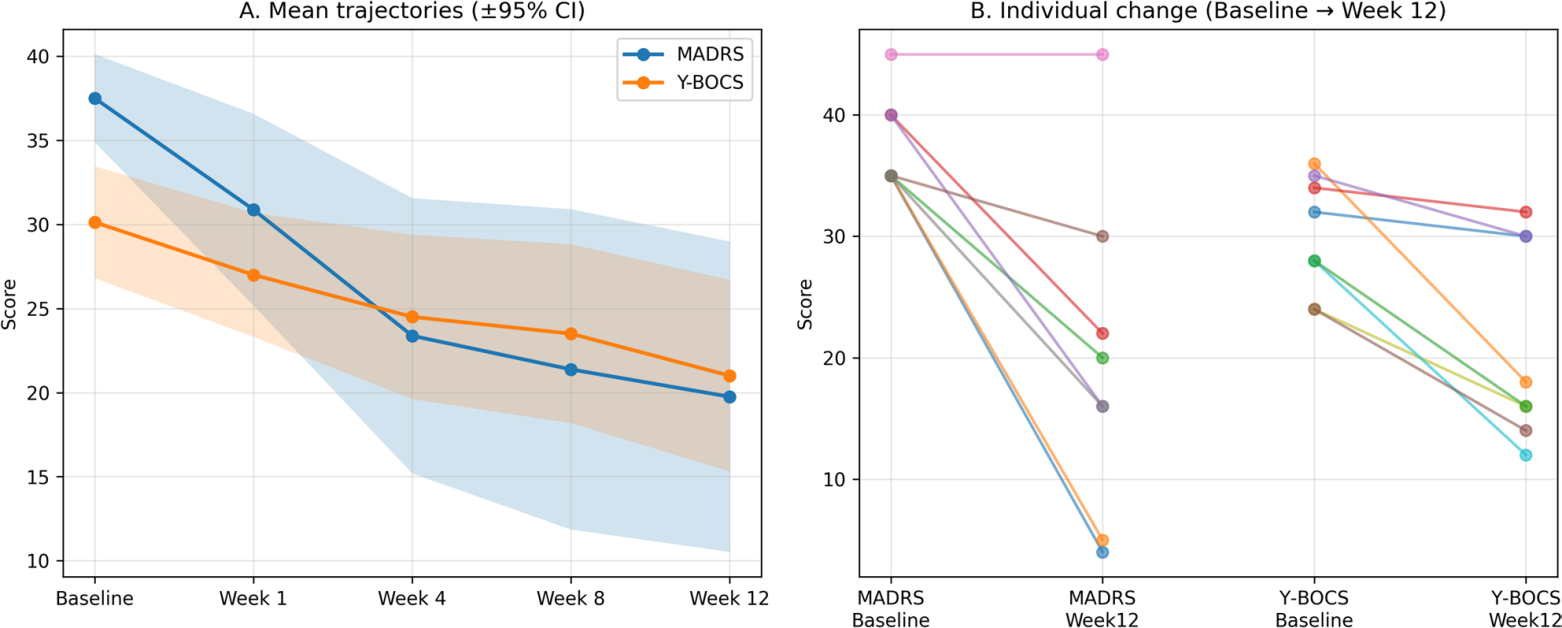
Symptom change over time and individual responses. (**A**) Mean MADRS and Y-BOCS scores across the 12-week observation period with 95% confidence intervals. (**B**) Individual patient changes from baseline to Week 12 for MADRS and Y-BOCS. In panel B, each line represents one participant

Four of eight participants (50.0%) met MADRS response criteria, and two of these (25.0%) achieved remission. Four of eight participants (50.0%) met the OCD response criterion. Wilson 95% CIs were [0.215, 0.785] for MADRS response, [0.071, 0.591] for MADRS remission, and [0.215, 0.785] for Y-BOCS response. Figure 2 summarizes symptom change over time and individual baseline-to-endpoint responses for MADRS and Y-BOCS.

### Responder versus non-responder patterns

Given the small sample size, no formal subgroup comparisons were performed. However, descriptively, patients with OCD response tended to show more progressive reduction in symptoms between Weeks 8 and 12, whereas non-responders generally displayed flatter Y-BOCS trajectories throughout follow-up. In contrast, antidepressant response often emerged earlier, mainly within the first month. These descriptive differences suggest that anti-obsessional benefit may require more sustained treatment exposure and may depend on clinical features not captured in the present sample, such as symptom dimension, cognitive rigidity, or capacity to engage in behavioral treatment.

### Tolerability and adverse events

No severe adverse events were documented. Four of the eight participants (50.0%) reported transient dissociative experiences, typically subsiding spontaneously within 20–30 min. One of the eight participants (12.5%) experienced moderate nausea. None withdrew because of adverse events.

## Discussion

To our knowledge, this is the first prospective case series to specifically examine repeated intranasal esketamine augmentation in patients with severe treatment-resistant obsessive–compulsive disorder (TR-OCD) and comorbid major depressive disorder (MDD). In this highly refractory sample, repeated intranasal esketamine was associated with clinically meaningful improvement in both depressive and obsessive–compulsive symptoms over 12 weeks, although the temporal pattern differed across symptom domains. Depressive symptoms improved earlier and more consistently, whereas anti-obsessional effects appeared more gradual and heterogeneous. This pattern is clinically relevant because it suggests that symptom domains may not respond in parallel and that short early observation periods may underestimate possible anti-obsessional benefit.

The antidepressant findings are broadly consistent with the established literature on esketamine in treatment-resistant depression and with more recent real-world data suggesting that response trajectories may extend beyond the earliest treatment window [17, 21]. In this respect, our results may be read in line with the REAL-ESK data, which indicate that some patients derive clinically meaningful benefit only after more sustained treatment exposure [21]. This is particularly relevant in OCD–MDD comorbidity, where improvement in depressive symptoms may precede improvement in obsessive-compulsive symptoms. Likewise, the work of Olivola et al. raises the possibility that constructs such as mentalization and emotional-cognitive rigidity may influence esketamine outcomes [20]. Although these variables were not assessed in our study, they may be especially pertinent to OCD and could partly account for the slower and less homogeneous anti-obsessional response observed here.

At the same time, the present findings should not be interpreted as proof of a specific anti-obsessional effect independent of nonspecific improvement. Because participants were selected on the basis of high baseline severity, regression to the mean is a plausible contributor to part of the observed score reduction. In the absence of a control group, randomization, or blinded assessment, we cannot formally disentangle true treatment-related change from natural fluctuation, expectancy effects, or the impact of repeated clinical contact. Accordingly, the present data should be interpreted as preliminary clinical observations rather than confirmatory efficacy evidence.

Baseline severity may also help explain the response pattern. Patients entering treatment with very high symptom scores have more statistical room for percentage improvement and may therefore be more likely to meet response criteria, while still remaining far from remission thresholds. This may be especially relevant in our sample, which was deliberately enriched for severe OCD and severe depressive symptoms. Thus, the relatively robust response rates should be interpreted alongside the still substantial endpoint burden in several patients, particularly for OCD symptoms.

In our series, four of the eight participants (50.0%) met MADRS response criteria, two of the eight (25.0%) achieved MADRS remission, and four of the eight (50.0%) met the predefined OCD response criterion. These figures compare favorably with prior exploratory work in OCD-spectrum esketamine treatment, while remaining broadly consistent with the notion that benefit is heterogeneous and still incompletely characterized in this population [23, 29]. Our observations also extend earlier reports summarized in the ketamine/esketamine literature for OCD, which has so far been dominated by small studies, single-dose intravenous designs, and isolated case-based evidence [11, 13, 23, 29]. In particular, the case report by Marcatili et al. described improvement in both depressive and obsessive-compulsive symptoms during intranasal esketamine treatment in comorbid TRD/OCD, but by design such evidence remains anecdotal and cannot establish reproducibility [29]. Our data modestly strengthen that signal by adding a prospective repeated-dose intranasal series, although still within the clear limits of an uncontrolled case series.

The slower and more variable reduction in obsessive-compulsive symptoms, compared with the more rapid antidepressant effect, also supports a more nuanced dual-action hypothesis. Rather than assuming a uniform effect across symptom domains, our findings suggest that esketamine may influence depressive and obsessive-compulsive dimensions through partially overlapping but temporally distinct mechanisms. This interpretation is conceptually compatible with prior work implicating glutamatergic dysfunction in both mood disorders and OCD, including cortico-striato-thalamo-cortical circuitry relevant to compulsivity [8, 9], and with the broader suggestion that esketamine’s clinical effects may extend beyond mood symptoms alone [22]. From a practical standpoint, this raises the possibility that sustained treatment exposure and perhaps combination with structured psychotherapy such as ERP may be necessary to obtain fuller anti-obsessional benefit.

Repeated intranasal esketamine was generally well tolerated in this sample. No serious adverse events were observed, and overall side effects were transient and manageable, suggesting that this approach is feasible in specialized psychiatric settings with appropriate monitoring. However, tolerability and feasibility should not be conflated with efficacy, and larger controlled studies will be needed before any firm clinical role can be assigned to intranasal esketamine in refractory OCD–MDD presentations.

Several limitations must be emphasized. The small sample size, observational design, and absence of randomization substantially restrict generalizability and preclude causal inference. The lack of a control condition limits interpretation of the observed changes and prevents formal separation of treatment effects from regression to the mean or nonspecific therapeutic factors. Follow-up was limited to 12 weeks, precluding conclusions about maintenance effects, relapse prevention, or the durability of anti-obsessional benefit. In addition, concomitant psychotherapy was not prospectively standardized or systematically quantified, which is particularly important in OCD and limits conclusions regarding the interaction between esketamine and behavioral treatment.

Future research should involve larger controlled prospective studies to clarify the magnitude and durability of benefit, identify predictors of response, and determine whether repeated intranasal administration offers meaningful advantages over other ketamine/esketamine routes or schedules in refractory populations. Trials that integrate structured ERP, neurocognitive phenotyping, and biomarkers of glutamatergic or circuit-level dysfunction may be especially informative in determining which patients are most likely to benefit and whether depressive improvement can be leveraged to enhance anti-obsessional treatment engagement.

## Conclusions

This prospective case series provides preliminary clinical data suggesting that repeated intranasal esketamine augmentation may be associated with improvement in both depressive and obsessive–compulsive symptoms in a subgroup of patients with severe TR-OCD and comorbid MDD. Depressive symptoms appeared to improve earlier, whereas obsessive-compulsive symptoms showed a slower and more heterogeneous trajectory. These findings help inform the ongoing evaluation of administration routes, dosing schedules, and treatment integration strategies for highly refractory OCD–MDD presentations, but they should be interpreted cautiously given the small sample, observational design, and lack of a control condition. Controlled prospective studies are needed to clarify efficacy, optimal treatment parameters, and the role of concurrent psychotherapy.

## Data Availability

All data generated or analyzed during this study are included in this published article. No additional datasets are available.

## Abbreviations

BZD: Benzodiazepine
CBT-ERP: Cognitive–behavioral therapy with exposure and response prevention
CGI-S: Clinical Global Impression–Severity
DLP: Dyslipidemia
HT: Hypertension
IBS: Irritable bowel syndrome
MADRS: Montgomery–Åsberg Depression Rating Scale
MDD: Major depressive disorder
NMDA: N-methyl-D-aspartate
OCD: Obsessive–compulsive disorder
SSRI: Selective serotonin reuptake inhibitor
TCA: Tricyclic antidepressant
TR-OCD: Treatment-resistant obsessive–compulsive disorder
Y-BOCS: Yale–Brown Obsessive Compulsive Scale
